# Phenotypic but no genetic adaptation in zooplankton 24 years after an abrupt +10°C climate change

**DOI:** 10.1002/evl3.280

**Published:** 2022-07-06

**Authors:** Antónia Juliana Pais‐Costa, Eva J. P. Lievens, Stella Redón, Marta I. Sánchez, Roula Jabbour‐Zahab, Pauline Joncour, Nguyen Van Hoa, Gilbert Van Stappen, Thomas Lenormand

**Affiliations:** ^1^ CEFE, CNRS, Univ Montpellier Univ Paul Valéry Montpellier 3, EPHE, IRD Montpellier 34293 France; ^2^ Marine and Environmental Sciences Centre (MARE), Faculty of Sciences and Technology University of Coimbra Coimbra 3004‐517 Portugal; ^3^ Aquatic Ecology and Evolution, Department of Biology University of Konstanz Konstanz 78464 Germany; ^4^ Department of Wetland Ecology Estación Biológica de Doñana‐CSIC Sevilla 41092 Spain; ^5^ Departamento de Biología Vegetal y Ecología, Facultad de Biología Universidad de Sevilla Sevilla 41012 Spain; ^6^ CNRS, Université de Rennes 1, ECOBIO (écosystème, biodiversité, évolution) ‐ UMR 6553 Rennes 35042 France; ^7^ Department of Coastal Aquaculture College of Aquaculture and Fisheries Can Tho University Can Tho Vietnam; ^8^ Laboratory of Aquaculture and Artemia Reference Center Ghent University Gent B‐9000 Belgium

**Keywords:** Additive genetic effect, climate change, microbiota, missing heritability, plasticity, resurrection ecology, thermal tolerance, transgenerational epigenetic effects

## Abstract

The climate is currently warming fast, threatening biodiversity all over the globe. Populations often adapt rapidly to environmental change, but for climate warming very little evidence is available. Here, we investigate the pattern of adaptation to an extreme +10°C climate change in the wild, following the introduction of brine shrimp *Artemia franciscana* from San Francisco Bay, USA, to Vinh Chau saltern in Vietnam. We use a resurrection ecology approach, hatching diapause eggs from the ancestral population and the introduced population after 13 and 24 years (∼54 and ∼100 generations, respectively). In a series of coordinated experiments, we determined whether the introduced *Artemia* show increased tolerance to higher temperatures, and the extent to which genetic adaptation, developmental plasticity, transgenerational effects, and local microbiome differences contributed to this tolerance. We find that introduced brine shrimp do show increased phenotypic tolerance to warming. Yet strikingly, these changes do not have a detectable additive genetic component, are not caused by mitochondrial genetic variation, and do not seem to be caused by epigenetic marks set by adult parents exposed to warming. Further, we do not find any developmental plasticity that would help cope with warming, nor any protective effect of heat‐tolerant local microbiota. The evolved thermal tolerance might therefore be entirely due to transgenerational (great)grandparental effects, possibly epigenetic marks set by parents who were exposed to high temperatures as juveniles. This study is a striking example of “missing heritability,” where a large adaptive phenotypic change is not accompanied by additive genetic effects.

**Impact Summary**

Adaptation is often rapid when environments change quickly, but for climate warming little evidence is available. Many studies report no genetic responses due to preexisting developmental plasticity, whereas others point toward epigenetics and microbiota effects. In this study, we take advantage of a natural experiment to study all of these effects. We use a set of coordinated experiments and a “resurrection ecology” approach, reviving resting eggs of brine shrimp up to 100 generations after their introduction from a temperate to a tropical saltern. We find that heat adaptation occurs, but heritability is largely “missing.” Plasticity and microbiota do not play a role in the increased thermal tolerance either, suggesting that only transgenerational (great)grandmaternal effects are involved. This finding prompts us to carefully reconsider the relative importance of the different possible mechanisms by which phenotypic change can occur, especially in response to temperature variation.

Understanding how biodiversity responds to global warming and anticipating whether species will be able to adapt quickly enough to keep pace with the projected changes have become major scientific challenges (Hoffmann and Sgrò 2011). Although rapid genetic adaptation to novel human‐made environmental changes—pollution, pesticides, antibiotics—has been extensively documented (Hendry et al. 2017), much less has been observed for climate warming (Gienapp et al. 2008; Hoffmann and Sgrò 2011; Franks and Hoffmann 2012; Merilä and Hendry 2014; Stoks et al. 2014). This discrepancy might be due to the (as yet) modest climate change or to the fact that many preexisting mechanisms are already in place in most species to cope with the current range of climatic variation.

Theoretically, several mechanisms may cause a phenotypic response to climate warming (Gienapp et al. 2008; Franks and Hoffmann 2012). First, organisms may genetically evolve to better tolerate high temperatures, and this process may extend their tolerance outside their current thermal niche. They may also phenotypically adjust to these changes using preexisting plastic responses, within (Lande 2015; Chevin and Hoffmann 2017) or across generations (maternal effects, transgenerational epigenetic effects [Auge et al. 2017; Lind and Spagopoulou 2018]). Finally, they may also benefit from symbionts/microbiota adapted to these new conditions (Nougué et al. 2015; Vannier et al. 2015; Frankel‐Bricker et al. 2020), without adapting to these conditions themselves. These sources of variation are mutually nonexclusive and can interact in ways that are difficult to disentangle. For instance, maternal effects may be mediated by transmitted symbionts, epigenetic marks, or maternal plastic responses (Palumbi et al. 2014; Schlichting and Wund 2014; Vannier et al. 2015).

We investigated whether species could adapt beyond their climatic niche in the wild, with the aim of disentangling these different effects. We used a resurrection ecology approach to assess the thermal adaptive potential of natural populations of the brine shrimp *Artemia franciscana* over 24 years (about 100 generations) following an abrupt climatic shift (Lenormand et al. 2018). In the early 1980s, *A. franciscana* from San Francisco Bay, USA (hereafter SFB) were introduced into Vinh Chau solar saltern, Vietnam (hereafter VCH), where mean (air) temperatures are +10°C higher (Clegg et al. 2000; Frankenberg et al. 2000). This far exceeds the worst IPCC climate warming scenario for the 21st century (RCP8.5 Model predicts +6°C [IPCC 2013]), yet the brine shrimp have thrived (Van Hoa 2014), and show phenotypic adaptation to high temperatures (Clegg et al. 2000; Kappas et al. 2004). Indeed, VCH *Artemia* are now commonly used to inoculate other (sub)tropical salterns. We used a series of coordinated experiments to determine the extent to which the introduced *Artemia*’s phenotypic adaptation to higher temperatures resulted from genetic changes, preexisting plastic responses, transgenerational effects, or the effect of locally adapted microbiota (Fig. S1 presents expectations).

We compared the temperature tolerance of an ancestral population from SFB (cysts collected in 1984; hereafter SFB_84_) with that of two populations from VCH (cysts collected in 1997 and 2008; hereafter VCH_97_ and VCH_08_). We resurrected an F0 generation from each population and kept them at a standardized lab temperature (intermediate between temperatures at VCH and SFB, although closer to the latter), thus removing plastic maternal effects. We then measured juvenile survival in the F1 generation in common garden experiments under temperatures mimicking daily thermal conditions in SFB and VCH (hereafter *T*_SFB_ and *T*_VCH_). This experiment was repeated several times as the “control” treatment in the juvenile acclimation, parental acclimation, and microbiota experiments (see below). Very consistently in these controls, VCH populations raised in the laboratory showed increased juvenile survival compared to the original SFB_84_ population, but only when exposed to a VCH climate (meta‐analysis χ^2^(1) = 9.6, *P* = 0.002 at *T*_VCH_ and χ^2^(1) = 0.8, *P* = 0.38 at *T*_SFB_; Fig. 1A solid points). The VCH populations are thus phenotypically adapted to high temperatures, consistent with previous studies (Clegg et al. 2000; Frankenberg et al. 2000; Kappas et al. 2004), and this is not due to direct plastic maternal effects (because all F0 females were raised in the same conditions) or to different resource allocation of VCH females to their offspring—as the effect is specific to *T*_VCH_. Furthermore, VCH_08_ juveniles had significantly higher survival at *T*_VCH_ than VCH_97_ juveniles (post hoc *z* = 3.1, *P* = 0.002; Fig. 1A), so phenotypic adaptation increased over time in VCH.

**Figure 1:**
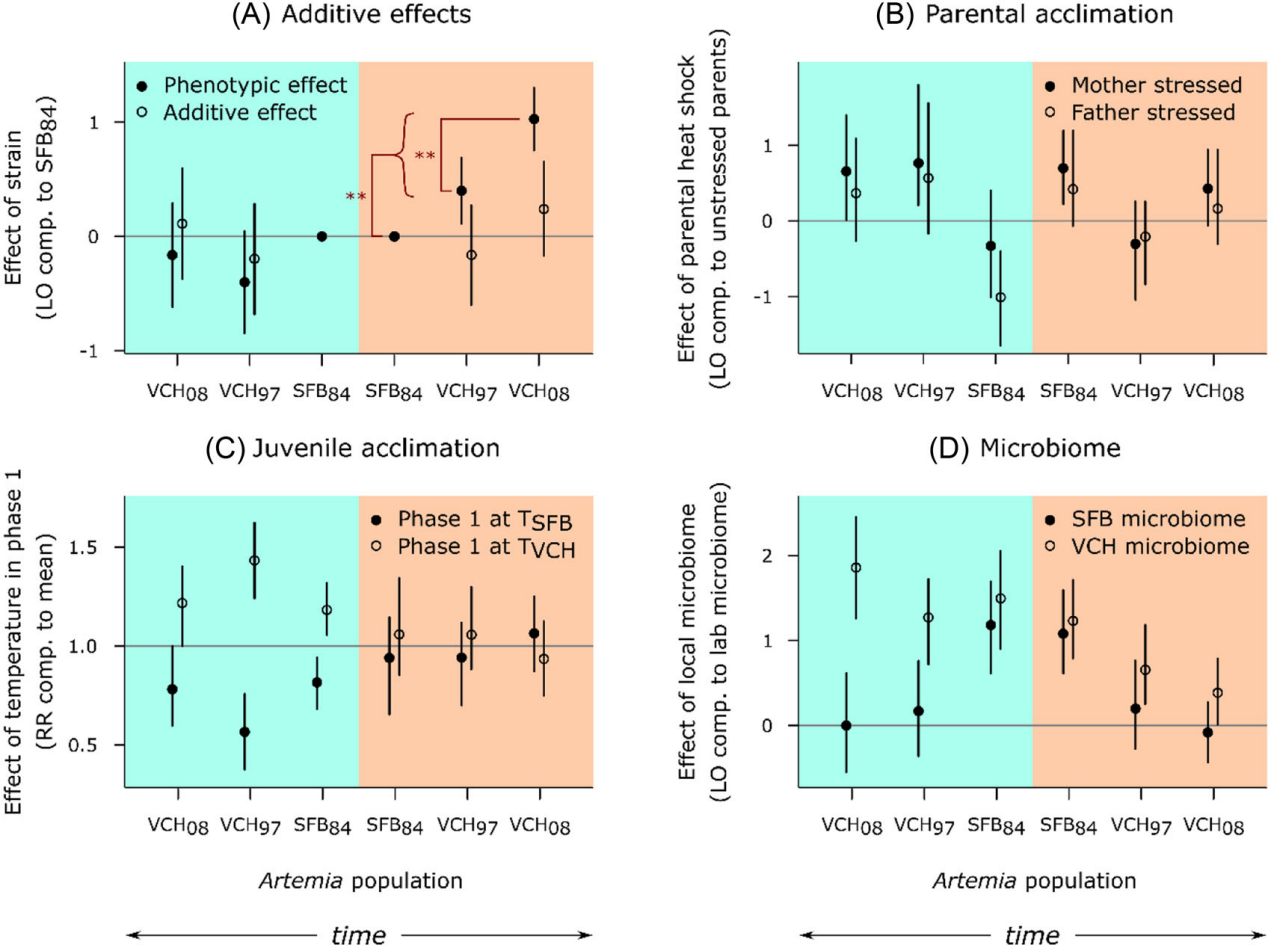
Disentangling the effects of genetics, parental acclimation, juvenile acclimation, and microbiome on phenotypic adaptation to high temperatures. Blue and orange backgrounds represent assays run at *T*_SFB_ and *T*_VCH_, respectively. The gray line corresponds to a lack of effect; bars are CIs. To maintain clarity, only significant differences relevant to the phenotypic adaptation to high temperature in VCH are shown; for other *P*‐values, see Table S2. This figure is related to Figure S1, which presents some simple scenarios, and to Figures S5–S9, which show the raw data. LO = log odds ratio of survival; RR = relative risk of survival; comp. = compared. (A) Survival of the VCH strains compared to the ancestral SFB_84_, when mothers belonged to the own population (solid points) and to an SFB reference population (“crossed” populations, empty points). The “0” points for SFB_84_ are included for reference. (B) Difference in survival between the second and first clutches, when parents were exposed to high temperature between clutches 1 and 2. The effect of the second clutch itself (which may have differed in survival compared to the first) is controlled for using the second versus first clutch effect observed for the unexposed control parents. (C) Survival in Phase 2, after exposure to *T*_SFB_ or *T*_VCH_ in Phase 1. Here, “mean” is the mean survival in Phase 2 for each strain. (D) Survival after inoculation with a local microbiome, compared to survival with the reference lab microbiome.

A second, crucial step was to determine whether this increased performance resulted from genetic changes. If so, VCH males should be able to transmit at least part of this increased performance to their progeny. We crossed SFB_84_, VCH_97_, and VCH_08_ males with reference SFB females from a stock cultured for over 2 years under standardized experimental conditions (see *Methods*). This cross removed any maternal and (great)‐grandmaternal effects that might have contributed to the observed phenotypic variation. Assuming that adaptation to a warmer climate is a polygenic trait, we expect roughly half of the additive genetic effects to be transmitted through males. We would therefore expect to see increased performance at *T*_VCH_ for the crossed VCH_97_ and VCH_08_ populations, but not for the crossed SFB_84_ population in the same juvenile survival test. Despite the strong phenotypic change observed in the uncrossed F1s, survival was not significantly different in juveniles from crossed SFB_84_, VCH_97_, and VCH_08_ populations in either temperature treatment (*P* = 0.44 for a population‐level difference at *T*_SFB_; *P* = 0.16 at *T*_VCH_; Table S2; Fig. 1A open points). This means that the increased performance of VCH *Artemia* at *T*_VCH_ did not result from additive genetic effects, a conclusion supported by post hoc analyses. If the increased performance was caused by genetic changes, it would be almost entirely recessive in our crossed populations (dominance level estimated at 0.10; Fig. S2). To obtain this overall estimate, a majority of alleles conferring thermal tolerance would need to be very recessive (i.e., with dominance levels of 0.1 or less). An analysis of plausible selection responses given the number of generations and population size confirmed that beneficial alleles with these dominance levels would not be expected to sweep quickly enough to explain the rapid phenotypic change we observed (Fig. S4). Therefore, (nuclear) genetic effects are unlikely to explain the magnitude of increased thermal tolerance at VCH.

Instead, this phenotypic change may have resulted from (i) maternal genetic effects, notably through mitochondrial evolution, or (ii) plastic grandmaternal (or earlier great‐grandmaternal, etc.) effects, for example, the transmission of epigenetic marks acquired in VCH. We investigated the possibility of mitochondrial evolution by sequencing the mitochondrial genome of 10 individuals from SFB_84_ and VCH_08_, as well as sequencing pooled cysts from VCH collected at eight dates between 1984 and 2008. SNP analyses show that mitotype frequencies were remarkably stable over that period, excluding a role for adaptation via the mitochondrial genome (Fig. 2, *Methods*). Hence, it is most likely that the VCH populations have not adapted genetically to higher temperatures. This finding is surprising, but other studies on adaptation to climate warming have also reported an absence of genetic response (Gienapp et al. 2008; Franks et al. 2014; Merilä and Hendry 2014). Frankenberg et al. (2000) also showed that VCH *Artemia* populations (hatched from field cysts collected in 1994) had increased survival at high temperature (compared to SFB cysts collected in 1978), but this increased performance was not apparent in later laboratory generations. Such a finding could result from transgenerational effects, supporting our third hypothesis of plastic (great‐)grandmaternal effects. Grandmaternal effects are also supported by the study of Norouzitallab et al. (2014), who report transgenerational epigenetic effects on thermal tolerance in laboratory *A. parthenogenetica*, which were transmitted up to the F3 generation.

**Figure 2:**
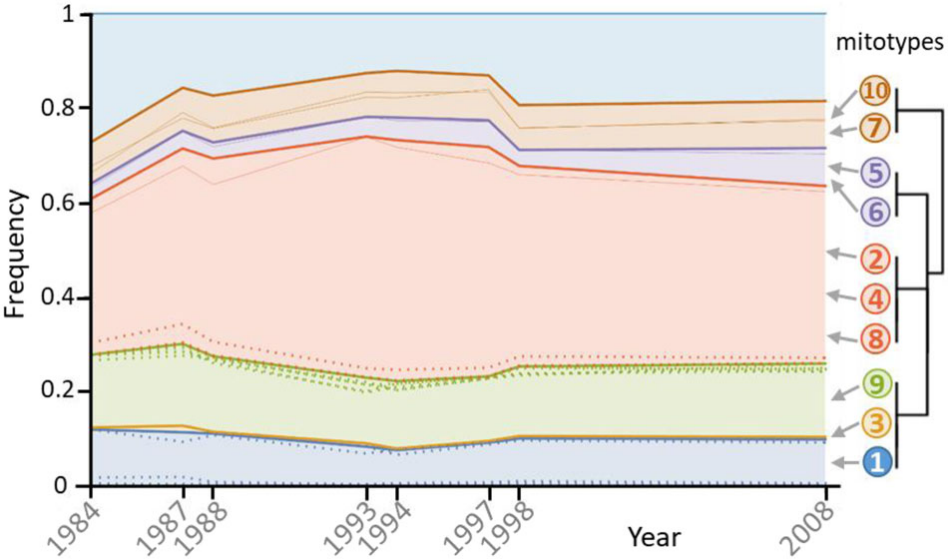
Mitotype frequency variation through time. Sampled years are shown on the *x*‐axis; the *y*‐axis expresses cumulative frequency. The relationship between the different mitotypes (based on shared‐SNP, methods) is shown by the dendrogram on the right. Mitotypes are shown with different colors; numbers identify the individual sequenced (1–5 from 1984 and 6–10 from 2008). The mitotypes’ frequency envelope is that of their most frequent shared‐SNP. Individuals 1, 3, and 9 do not have shared‐SNPs, and are therefore grouped on this dendrogram. Their frequency envelope is that of their most frequent private‐SNP. Thin lines represent other shared‐SNP frequencies within mitotypes. Dotted lines represent private‐SNPs within groups (only those reaching a frequency >1% are shown).

To investigate transgenerational effects on heat tolerance, we tested whether exposure of adult parents to *T*_VCH_ could influence progeny performance at *T*_SFB_ versus *T*_VCH_. If so, we would have a mechanism for the grandparental effects (provided they could be maintained for one more generation). We compared juvenile survival in clutches produced before and after exposing their parents to high temperatures (“Parental acclimation” experiment). We exposed the mother, the father, or neither parent. Comparing within the same family controlled for biases resulting from differential mortality of parents; comparisons with families where neither parent was exposed controlled for a second clutch effect. Results showed no significant differences in survival between clutches from the different parental treatments at *T*_SFB_ or *T*_VCH_ for any *Artemia* population (0.08 ≤ *P* ≤ 0.36 for a population, parental treatment, or interaction effect at *T*_SFB_; 0.15 ≤ *P* ≤ 0.41 at *T*_VCH_; Table S2; Fig. 1B), indicating that thermal exposure in adult parents does not detectably improve the thermal tolerance of their progeny. This experiment suggests that epigenetic marks are not set in adults in the time window preceding ovoviviparous clutch production. It is possible that epigenetic marks are only set when cysts are produced, although this is contradicted by Norouzitallab et al. (2014). More likely is that epigenetic marks are set during the juvenile development of the parents (or grandparents, etc.) (Norouzitallab et al. 2014; Donelson et al. 2018). The imprint may be set early during meiosis in the female germ line, which occurs during juvenile development (Lenormand et al. 2016). Indeed, the epigenetic effects referenced above were found after exposing juvenile *A. parthenogenetica* to a heat shock (Norouzitallab et al. 2014). Similar mechanisms are likely to operate in the sexual *A. franciscana*, but confirming these effects would be very challenging: exposing juveniles to environmental stress usually causes some mortality (e.g., in our results), making it difficult to exclude selection for stress‐tolerant genotypes in the treatment compared to the control. An alternate explanation is that our heat stress was not sufficiently stressful to elicit an epigenetic response in the heat‐tolerant VCH populations. This might be supported by a weak trend in the expected direction for SFB_84_. Although insufficient to drive a significant interaction effect, it might reveal that heat conditioning has different biological significance for populations with different heat tolerance. If so, it would reinforce the conclusion that epigenetic effects are at play.

Next, we investigated whether *Artemia* have a developmental plasticity response to the thermal environment. Such a plastic response would not be sufficient to explain the phenotypic effects that we observed, because these experiments did not include an acclimation phase before measurement. However, if plastic adjustment to cope with high temperatures preexisted in SFB, or evolved in VCH, this would help explain the lack of genetic change in VCH. This possibility is reinforced by previous studies in both juvenile and adult *Artemia*, which demonstrated plastic responses to thermal stress through the induction of heat shock proteins (Clegg et al. 2000; Frankenberg et al. 2000). To investigate this possibility, we exposed 5‐day‐old juveniles to *T*_SFB_ or *T*_VCH_ for 2 days, and then tested whether pre‐exposure increased performance in each environment (“Juvenile acclimation” experiment) during the same age window used for the other experiments. Strikingly, we found that early exposure to *T*_VCH_ did not increase juvenile survival at *T*_VCH_ in any of the *Artemia* populations (*P* ≥ 0.62 for an effect of pre‐exposure or its interaction with population; Table S2; Fig. 1C). In contrast, pre‐exposure to *T*_VCH_ significantly increased survival at *T*_SFB_ (*P* < 0.0001 for a pre‐exposure effect; Table S2; Fig. 1C) for all three *Artemia* populations (*P* = 0.31 for an interaction with population; Table S2), indicating that there is indeed a plastic response (e.g., activation of heat shock proteins; Clegg et al. 2000; Frankenberg et al. 2000). However, this plasticity does not confer improved performance at *T*_VCH_, so it is unlikely to play a major role in the thermal adaptation at VCH.

Last, we investigated whether performance at *T*_SFB_ and *T*_VCH_ could be affected by the presence of microbiota adapted to those climates (“Microbiota” experiment). In corals, for example, the temperature niche is controlled by that of their symbionts (Littman et al. 2010). *Artemia* host many gut bacteria that are essential for the proper digestion of unicellular algae, their main food source. Adaptation of this microbiota to high salinity has been shown to determine their host's salinity niche (Nougué et al. 2015). Hence, it is possible that *Artemia*’s thermal niche is controlled in part by the thermal niche of its microbiome. Such a finding would also help explain the lack of genetic change in VCH. To evaluate this possibility, we investigated the thermal tolerance of axenic *Artemia* from SFB_84_, VCH_97_, and VCH_08_ populations inoculated with microbes sampled from live *Artemia* in SFB, VCH, or our reference laboratory cultures. If microbes contribute to thermal tolerance, we would expect VCH microbes to increase juvenile survival at *T*_VCH_, but not *T*_SFB_, whereas SFB microbes should increase survival at *T*_SFB_ but not *T*_VCH_ (Fig. S1). We did not find this pattern. Instead, we found that having microbes from VCH increased survival for all *Artemia* populations at both *T*_SFB_ and *T*_VCH_, whereas having lab microbes decreased survival in all circumstances (*P* = 0.003 for an interaction between population and microbiome at *T*_SFB_; *P* = 0.001 at *T*_VCH_; Table S2; Fig. 1D). Hosting VCH microbes appears to simply be better than hosting lab microbes. For the SFB microbes, we found that they conferred the same survival as VCH microbes in SFB_84_ but were equally poor as the lab microbes for VCH populations. Hence, our results are consistent with the idea that (i) microbes have a large impact on survival, (ii) microbes from our three stocks are different, and (iii) their effect depends on the *Artemia* population. We did not find any indication that the microbes play a role in thermal adaptation. Interestingly, we found that *Artemia* had no problems when exposed to microbiota from a tropical climate: they are available, and there is no need to specifically adapt to them (as SFB_84_ performed equally well with VCH microbes). All our findings are consistent with a loss of function in the laboratory microbes, and by a loss of ability of the Vietnamese *Artemia* to benefit from their ancestral SFB microbes.

In summary, we found no indication of genetic adaptation to increased temperature in a field situation that should a priori be very favorable for the evolution of thermal tolerance (Reznick and Ghalambor 2001): a large and isolated sexual population without initial bottleneck, exposed to a large and abrupt environmental shift over 100 generations. However, we did find a phenotypic difference when testing individuals whose grandmothers were exposed to high temperatures, and this difference was larger for the VCH_08_ population than for VCH_97_. These findings suggest that VCH *Artemia* have higher heat tolerance due to transgenerational effects, and that these effects increased through time, for example, by being better maintained through generations in more recent Vietnamese populations. Such effects are not entirely unexpected, as they are found more often in short‐lived, dispersal‐limited organisms, for juvenile traits, and in conditions where environmental variation is predictable over several generations (Yin et al. 2019). Our experiments point toward juvenile stress as the key trigger of transgenerational thermal tolerance. Further work is necessary to confirm this, but would be very challenging for the sexual *A. franciscana*. By comparing the survival of siblings produced before and after exposing parents to heat stress, our parental acclimation experiment excluded confounding effects of genetic change. In contrast, exposing juveniles to environmental stress would causes some mortality, making it difficult to exclude selection for stress‐tolerant genotypes operating in the treatment compared to the control. In consequence, indirect evidence for transgenerational heat tolerance by excluding all other factors remains our most powerful tool. The presence of transgenerational effects could explain the lack of genetic changes in VCH: transgenerational effects could keep the population phenotype close to a thermal optimum, thereby reducing directional selection and genetic changes. Such interference with genetic adaptation has been found in many studies reporting within‐generation plasticity (Gienapp et al. 2008; Merilä and Hendry 2014), but the transgenerational mechanism suggested here is much less documented. This may be because transgenerational effects are difficult to detect. The resurrection ecology approach is among the most powerful methods to study adaptation to climate change (Orsini et al. 2013; Lenormand et al. 2018; Nogués‐Bravo et al. 2018; Weider et al. 2018), but the possibility to perform crosses between the evolved and nonevolved populations turned out to be crucial. Without such crosses, we would likely have concluded that genetic adaptation had taken place (as in Geerts et al. 2015; Yousey et al. 2018).

Our study provides a striking example of adaptation involving traits whose heritability is largely “missing” (Trerotola et al. 2015), and where the phenotypic response is not caused by developmental plasticity. Overall, this work represents one of the most complete studies jointly addressing the different factors associated with thermal adaptation in the wild, namely, genetic effects, epigenetic effects, plasticity, and microbiota. In particular, we find striking putative transgenerational effects. The effects are large compared to other studies (Jeremias et al. 2018; Yin et al. 2019; Sánchez‐Tójar et al. 2020), and contrast with the absence of adaptive genetic and within‐generation plastic effects. This study prompts us to carefully consider the different mechanisms by which phenotypic change can occur, and their relative importance. It may also suggest that epigenetic responses, in addition to plastic responses, are particularly efficient and important when coping with environmental fluctuations. This may be a general aspect of adaptation to temperature, as it fluctuates constantly, and at different time scales.

## Methods

Experiments were performed with three populations of *A. franciscana*: one from San Francisco Bay (SFB), USA, collected in 1984 (SFB_84_); a second from Vinh Chau (VCH) saltern, Vietnam, collected in 1997 (VCH_97_); and a third, also from VCH, collected in 2008 (VCH_08_).

Seasonality is very limited in VCH. South Vietnam is characterized by a tropical climate without winter. The temperature tends to increase toward the end of the dry season, when the water eventually exceeds 35°C. This is associated with a rapid decline in both algae and *Artemia* populations. During the wet season, ponds are washed out and salinity drops below the level where *Artemia* populations are sustainable. Ponds are re‐inoculated each year using cysts from the previous year. The population dynamics in the field are limited by food supply (availability of unicellular algae). There are nearly four generations per year (i.e., ∼54 between 1984 and 1997 and ∼100 between 1984 and 2008).

### DECAPSULATION AND HATCHING OF THE CYSTS

The parental generation of experimental individuals (see below) was hatched from dormant cysts. Cyst decapsulation and hatching protocols were modified from Bengtson et al. (1991). Cysts were rehydrated in deionized water (2–3 h). After rehydration, cysts were decapsulated by a 10‐min exposure to a sodium hypochlorite solution (2.6%), then rinsed with running water (10 min) and deionized water (5 min). Decapsulated cysts were incubated for 48 h at 28°C (±1°C), with constant light and aeration, in a 5‐g/L salinity medium (see below). After emergence, first‐instar nauplii were moved to 23°C (±1°C) and natural light conditions. Salinity was gradually increased to 80–90 g/L over 8–9 days. This procedure was performed independently for the three different *Artemia* populations.

### BASELINE EXPERIMENTAL CONDITIONS

Throughout the preparation and execution of the experiments, *Artemia* were kept in an 80–90 g/L saline medium prepared by diluting field‐collected concentrated brine (280 g/L, Camargue Pêche, France) from Aigues‐Mortes saltern with deionized water. Organisms were fed a solution of *Tetraselmis chuii* algae (Fitoplankton marino, Spain), prepared by dissolving 1 g of lyophilized algae in 1 L of deionized water (about 6.8 × 10^9^ *T. chuii* cells/L). Stock individuals were fed ad libitum. Food was added daily (1 mL of algae/per group of juveniles/day; and 1 mL of algae/per couple/day) before exposure and three times a week (2 mL of algae/group of juveniles/2 days) during exposure to the temperature treatments. Unless specifically mentioned, individuals were kept at 23°C (±1°C), under natural light conditions. Juvenile survival tests were all performed in the dark in incubators and thermostatic chambers. Mortality was checked twice (5 and 10 days after the beginning of the treatment, i.e., midway through and at the end of the thermal treatment).

### SFB AND VCH TEMPERATURE REGIMES

The same temperature regimes were applied in each experiment. Two temperature cycles were used: (i) cycle of temperatures based on the air temperatures from SFB (*T*_SFB_): 16°C (2 h); 22°C (8 h); 27°C (4 h); 22°C (8 h); 16°C (2 h); and (ii) cycle of temperatures based on the air temperatures from VCH saltern (*T*_VCH_): 26°C (2 h); 32°C (8 h); 37°C (4 h) for experiment 2 and 35°C (4 h) for the remaining experiments; 32°C (8 h); 26°C (2 h).

### EXPERIMENT 1: MICROEVOLUTION/ADAPTATION

This experiment was performed to measure the additive genetic effect of thermal adaptation, removing maternal lineage effects. For this experiment, we collected virgin females from a laboratory population of *A. franciscana* from SFB, hatched from cysts collected in 2003 (SFB_03_). The SFB_03_ population was maintained in the laboratory for over 2 years, so it was well acclimated to the standard laboratory temperature conditions (23°C ± 1°C). For SFB_84_, VCH_97_, and VCH_08_, we hatched individuals from field cysts. Before individuals reached sexual maturity, their sex was assigned based on sexual dimorphism. After maturity, males from the three study populations of *Artemia* were mass crossed (animals divided into four replicates) with the virgin stock females (SFB_03_) to produce an F1 generation. Starting 24 h after the first nauplii were seen, we collected daily batches of nauplii from the mass crosses. This ensured that the organisms used in each replicate were born within the same period. Newborn nauplii from each cross were maintained in 50‐mL Falcon tubes (maximum 30 nauplii per tube) filled with 30 mL of brine solution for a period of 7 days. After 7 days, all meta‐nauplii from the same cross were mixed and then separated into replicate groups of 10 individuals. Each group was placed in a 50‐mL Falcon tube filled with 30 mL of brine solution, and exposed to *T*SFB or *T*VCH for 10 days (7th to 17th day). A total of 30–32 groups per population were exposed to each cycle of temperatures (1830 individuals in total).

### EXPERIMENT 2: PARENTAL ACCLIMATION

This experiment was designed to investigate the possibility that thermal exposure in the parents could influence juvenile performance at high temperature. Individuals were hatched from SFB_84_, VCH_97_, and VCH_08_ field cysts. Before they reached sexual maturity, their sex was assigned based on sexual dimorphism. After maturity, single pairs of males and females from each population were isolated in 50‐mL Falcon tubes filled with 30 mL of brine solution to produce an F1 generation. We collected the first brood of nauplii produced by each parental couple. Each brood of nauplii was isolated from their parents after confirming, under a stereomicroscope, that the female ovisac was empty. In this way, we ensured that the organisms used in each replicate were born within the same period. Immediately after the first clutch (CL_1_) was born, the parents were separated, and one of three treatments was applied: (i) mother exposed to high temperature; (ii) father exposed to high temperature; and (iii) control (none exposed to high temperature). The “high temperature” treatment consisted of 8 h at 35°C (±1°C) in the dark. Afterward, the couples were put back together to produce a second clutch (CL_2_), which we collected in the same way. Newborn nauplii were kept in 50‐mL Falcon tubes (maximum 30 nauplii per tube) filled with 30 mL of brine solution for a period of 7 days. After 7 days, meta‐nauplii from each family were separated into groups of 10 individuals and placed in 50‐mL Falcon tubes filled with 30 mL of brine solution and exposed to *T*_SFB_ or *T*_VCH_ for 10 days (7th to 17th day). For the SFB_84_ population, we obtained 54 couples who produced a first and a second clutch (other couples were discarded). We used on average 83 offspring per couple (range 40–110, grouped in tubes of 10 individuals), evenly split between the first and second clutch and *T*_SFB_ and *T*_VCH_ (for a total of 4470 offspring tested). Couples were evenly assigned a treatment (control, mother, or father stressed between clutch 1 and 2). For VCH_97_, for the same design, we had 48 couples, 73 offspring per couple on average (range 40–110), for a total of 3500 offspring tested. For VCH_08_, for the same design, we had 56 couples, 84 offspring per couple on average (range 50–110), for a total of 4720 offspring tested. The overall experiment involved 12,690 individuals.

### EXPERIMENT 3: JUVENILE ACCLIMATION

This experiment was conducted to study if very early exposure of the organisms to a thermal regime would increase their performance as juveniles under the same regime. Individuals were hatched from SFB_84_, VCH_97_, and VCH_08_ field cysts. Before individuals reached sexual maturity, their sex was assigned based on sexual dimorphism. After maturity, single pairs of males and females from the same population were placed in 50‐mL Falcon tubes filled with 30 mL of brine solution to produce an F1 generation. We collected newborn nauplii from the parental couples. Each brood was isolated after confirming, under a stereomicroscope, that the female ovisac was empty. In this way, it was ensured that the organisms used in each replicate were born within the same period. Newborn nauplii were counted and separated into 50‐mL tubes containing 30 mL brine solution (maximum 30 nauplii per tube). Nauplii were then maintained under the same conditions of light, food, and temperature as the parents for a period of 5 days. After 5 days, meta‐nauplii entered the experiment, which was divided into two phases (P_1_ and P_2_). At day 5, a first temperature regime was applied for 2 days (P_1_). Meta‐nauplii from each family were separated into 50‐mL Falcon tubes (maximum 30 nauplii per tube) filled with 30 mL of brine solution and assigned to either *T*_SFB_ or *T*_VCH_. Broods were discarded whenever it was impossible to obtain two replicates (one per temperature regime) with a minimum of 10 individuals each. After this first phase (P_1_), mortality was checked, and surviving meta‐nauplii were separated into groups and placed into 50‐mL Falcon tubes (no more than 14 individuals per falcon) filled with 30 mL of brine solution. Broods were discarded whenever it was impossible to obtain two replicates (one per temperature regime) with a minimum of five individuals each. Meta‐nauplii from each population and temperature regime were again assigned to *T*_SFB_ or *T*_VCH_ for the second phase (P_2_). Hence, different individuals were exposed to different temperature histories: *T*_SFB_ → *T*_SFB_, *T*_SFB_ → *T*_VCH_, *T*_VCH_ → *T*_SFB_, *T*_VCH_ → *T*_VCH_. Survival during this second phase was recorded for a period of 10 days (7th to 17th day). Overall, 48.8 (SD 12.0) groups were used per temperature history (P_1_ → P_2_) and population combination (survival of 5315 individuals assayed in total).

### EXPERIMENT 4: MICROBIOTA

This experiment was designed to investigate whether exposing organisms to microbiota adapted to different climates lent their hosts different performance in those climates. SFB_84_, VCH_97_, and VCH_08_ field cysts were rehydrated in sterile deionized water (2–3 h). After rehydration, cysts were decapsulated by a 10‐min exposure to a sodium hypochlorite solution, then rinsed with deionized water (10 min) and sterile deionized water (5 min). Decapsulated cysts were then incubated for 3 days at 28°C (±1°C) and under constant light, in sealed bottles containing 400 mL autoclaved brine solution (5 g/L). This procedure has been shown to be very effective in producing axenic *Artemia* (Nougué et al. 2015). After emergence, first‐instar nauplii were placed at 23°C (±1°C) under constant light and fed with sterilized *T. chuii* solution. This procedure was performed independently for the three different populations. Salinity was gradually increased to 80–90 g/L over 8–9 days. When salinity reached 40 g/L, nauplii from each population were separated into three groups and inoculated with (i) microbiota from SFB, (ii) microbiota from VCH, or (iii) microbiota from containers in the laboratory. The microbiota initial inoculum solution was obtained by mixing crushed live adult individuals collected in 2017 in two sites in both Vinh Chau saltern (salinity 70 and 90 g/L, four individuals in 2 mL in eight replicates) and San Francisco Bay Estuary Field Station (salinity 70 and 130 g/L, four individuals in 1.5 mL in eight replicate tubes). These 2017 microbiota might differ from the original 1984 situation, but the thermal background did not significantly change between 1984 and 2008 and those microbial communities should reflect this climatic difference. These initial inoculant (mixing a low and high salinity tube in each case) were added to an axenic culture of each population (1 L, about 100 individuals, 80–90 g/L, 23°C) and incubated for over a month. Water from these cultures was used as an inoculation starter for the experiment for each corresponding population. For the laboratory microbiota, the inoculation starter was taken directly from nonaxenic cultures in the laboratory. Each inoculation bottle was filled with 400 mL of sterile deionized water and 100 mL of this microbiota starter solution. Sterilized *T. chuii* was added ad libitum. When individuals reached sexual maturity, 12 males and 12 females from each population and treatment were separated into new sterile containers and mass crossed to produce a F1 generation and kept under the same conditions as the stock. Newborn nauplii were checked daily. Each batch of nauplii was isolated within 24 h after the first nauplius was seen, to ensure that organisms used in the experiment were born within the same period. Newborn nauplii were separated into sterile 50‐mL tubes (maximum 30 nauplii per tube) containing 26 mL of sterile brine solution, 2 mL of microbiota starter solution, and 2 mL of autoclaved algae solution. Nauplii were then maintained under natural light at 23°C (±1°C) for a period of 7 days. After 7 days, all meta‐nauplii from the same treatment were mixed and separated into replicate groups of 10 individuals. Each group was placed in a sterile 50‐mL tube containing 26 mL of sterile brine solution, 2 mL of microbiota starter solution, and 2 mL of algae solution. To maintain the comparison with the other experiments, only the water was autoclaved to prepare the food solution for the rest of the experiment (i.e., not the lyophilized algae, which would have significantly altered the food source). Each replicate was exposed to *T*_SFB_ or *T*_VCH_ for 10 days (7th to 17th day). Overall, 87–103 groups (27–39 groups per microbiota treatment) per population were exposed to each temperature regime (5630 individuals in total). All feeding and transfers were performed under a laminar flow hood to prevent microbial contamination. During the experiment, the containers were closed to limit contamination, but not sealed to allow gas and oxygen exchange.

### STATISTICAL ANALYSES

We first analyzed the overall temperature tolerance of the VCH populations compared to the ancestral SFB_84_. To maximize our power to detect differences between populations, we pooled the “control” data from the acclimation and microbiome experiments. Specifically, we used the first clutches from the “Parental acclimation” experiment, the second clutches from the “Parental acclimation” experiment whose parents were not exposed to high temperature; the individuals from the “Microbiome” experiment who were inoculated with the lab microbiome; and the organisms from the “Juvenile acclimation” experiment who had undergone the same temperature regime in Phases 1 and 2. There are of course some small differences between these experiments (i.e., the “Juvenile acclimation” organisms had undergone a slightly longer exposure to the temperature regimes, the “Microbiome” organisms were cultured differently), but the meta‐analysis approach accounts for this additional variation. We used a multilevel meta‐analysis model (R Core Team n.d.; Viechtbauer 2010), and meta‐analyzed the two temperature regimes separately. Survival relative to the SFB_84_ population was taken as the response variable because it is the ancestral population. Effect sizes were obtained by fitting binomial models like those described below to the control data for each experiment, and extracting the log odds ratio of each VCH population relative to SFB_84_ (more details in Table S1). Standard errors extracted from the same models were used to weight the meta‐analysis. The full meta‐analysis model contained *VCH population* (VCH_97_ or VCH_08_) as a fixed effect, and *Experiment* as a random effect controlling for nonindependence within experiments. The significance of *VCH population* was then tested using likelihood ratio tests. Where relevant, post hoc Tukey tests were performed to compare the two populations.

To analyze the individual experiments, we used generalized linear mixed models (R Core Team n.d.; Bates et al. 2015), with the number of surviving versus dead *Artemia* in each replicate as the response variable (binomial response with logit link). The two temperature regimes were analyzed separately (i.e., the following was repeated for *T*_SFB_ and *T*_VCH_). First, we constructed a full model that included all the experimentally manipulated factors and their interactions. The “Additive genetic effects” models included only *Population*. The “Parental acclimation” models included *Population*, *Clutch* (a dummy variable, with the first clutch coded as “0” and the second clutch as “1”), and their interaction, and the interactions between these and the factor *Parental treatment*. By using the dummy variable and restricting *Parental treatment* to the interaction terms, we avoided generating spurious (and biologically impossible) estimates of the effect of *Parental treatment* on the first clutch. We also included a random *Family* term to group replicates collected from the same parental couple. In the “Juvenile acclimation” experiment, we analyzed the survival in Phase 2, which was conditional upon survival in Phase 1. The models included *Population*, *Temperature in Phase 1*, and their interaction, as well as a random *Family* term to group replicates collected from the same parental couple. For the “Microbiome” experiment, the models included *Population*, *Microbiome*, and their interaction. Where necessary, the full models were corrected for overdispersion by including an observation‐level random effect (Harrison 2015). Finally, the significance of the predictors was tested using likelihood ratio tests. For the “Parental acclimation” experiment, where we were only interested in the effects of *Population* and *Parental treatment* on the difference between the first and second clutch, we only tested the significance of the interaction terms.

### MITOCHONDRIAL GENOME SEQUENCING AND ANALYSES

To determine whether increased heat tolerance of the Vietnamese populations could be caused by mitochondrial genetic variation, we sequenced the full mitochondrial genome of 10 individuals (individuals 1–5 sampled in 1984, and individuals 6–10 sampled in 2008). We also sequenced pools of cysts sampled in Vinh Chau saltern (25 mg of cysts, about 6500 cysts per pool) from eight years (1984, 1987, 1988, 1993, 1994, 1997, 1998, and 2008). Three of these were replicated twice, with independent DNA extraction (1984, 1997, and 2008). For each sample, mitochondrial DNA was extracted using an Abcam ab65321 Mitochondrial DNA isolation kit, following the manufacturer's instructions. NGS libraries were constructed using a Nextera DNA flex illumina kit (ref 20018704) and sequenced (PE 150) on an Illumina NovaSeq 6000 (MGX platform, Montpellier).

For each sample, paired reads were mapped onto an *A. franciscana* reference sequence (NC_001620.1) with *bowtie2*, trimming 10 bases in 5'. Read duplicates were removed with *Picard MarkDuplicates*. Reads with a mapping quality over 20 and in proper pairs were kept with *samtools view*. The program *pysamstats* was used to get the raw percentage of each base and the total coverage at each position of the reference sequence. These steps were done twice, on the original reference genome and on a version that was cut in the middle and had the two parts reordered. This was done to avoid border effects and obtain a good mapping for the reference extremities of this circular genome. A dedicated *R* script was written to concatenate the *pysamstats* output files, keeping 50% middle positions of the two reference versions, to obtain two tables with all samples: one with the percentages of alternative bases at each position and one with the coverages. SNP calling was done using a dedicated Mathematica 10.1 (*Wolfram*) script. Genome coverage was ∼3000× on average for cyst pool samples (range 1000× to 6000×), and was ∼200× on average for individual samples (range 42× to 336×). Three regions showed a drop in coverage on the reference genome and were excluded from further analyses (region 1: 14045–14394; region 2: 14682–14835; region 3 15409–15806).

Forty variable positions were identified that distinguished the 10 sampled individuals. One of them was an ambiguous insertion of a variable number of Ts at position 1247, and was removed. Among the 39 remaining SNPs, seven were shared by at least two individuals and 32 were private to a single individual. The shared‐SNPs defined six nonambiguous haplotypes (hereafter “mitotypes”), three being characterized by a combination of at least two shared‐SNPs (individuals 7 and 10; individuals 5 and 6; individuals 2, 4, and 8) and three by the absence of shared‐SNPs (individuals 1, 3, and 9). The frequency envelopes of the former were obtained using the frequency of their most frequent shared‐SNP, whereas the frequency envelope for the latter was based on the frequency of their most frequent private‐SNP (as in the absence of recombination, the sum of the frequency of private SNPs cannot exceed that of shared SNPs within a mitotype).

The frequency of each of the 39 SNPs was estimated from the cyst pool‐seq data in eight separate years (Fig. 2). Frequencies at all shared and private SNPs were very highly correlated between replicates (*R* ^2^ = 0.995 for years 1984, 1997, and 2008), showing that the pool‐seq data provided very precise information (Fig. S3). Frequency data from consecutive years also showed very consistent frequency estimates (Fig. 2). The cumulative frequency of the six mitotypes identified represented ∼80% of the population. Other SNPs were identified in the dataset, but were not used as they could not be easily clustered or assigned to a mitotype due to the lack of important temporal frequency variation. Overall, the frequency pattern of the different mitotypes was remarkably stable, ruling out that the genetic composition of the mitochondrial population changed significantly over the study period. This, therefore, rules out that mitochondrial genetics explain the increased heat tolerance in the Vietnamese *Artemia* through time.

### CONFLICT OF INTEREST

The authors declare no conflict of interest.

### AUTHOR CONTRIBUTIONS

TL and MS conceptualized the idea of the study, performed supervision, and acquired funding. AJPC, SR, RJZ, and EJPL performed experiments. EJPL, TL, and PJ performed formal analysis. NVH, GVS, RJZ, and TL performed sampling. AJPC, TL, and EJPL wrote the original draft. All authors revised the manuscript.

### DATA ARCHIVING

Data and code are available on zenodo doi 10.5281/zenodo.6399057.

Associate Editor: S. Wright

## Supporting information


**Table S1**. Models used to generate effect sizes and variances for the meta‐analysis of phenotypic adaptation (solid points, Fig. 1A), which compared the overall temperature tolerance of VCH and SFB populations.
**Table S2**. Significance of the tested effects for the individual experiments. Temp., temperature; treatm., treatment.
**Figure S1**. Illustration of possible outcomes for the different experiments, with simple scenarios described next to the figures.
**Figure S2**. Post‐hoc analysis of additive effects. In order to analyse the phenotypic change through time, we computed the slope of the log odd score through time (taking SFB_84_ , VCH_97_, and VCH_08_ as time 0, 1, and 2, respectively).
**Figure S3**. SNP frequency data quality. SNP frequency was independently estimated twice for years 1984 (red), 1997 (orange), and 2008 (brown).
**Figure S4**. Frequency of a strongly beneficial recessive allele (*s* = 0.3) through time in a population of *N* = 10^7^ (panel A) or *N* = 10^6^ (panel B) individuals.
**Figure S5**. Survival data that was used in the meta‐analysis of phenotypic effect.
**Figure S6**. Raw survival data for the additive effect experiment. Each point represents one replicate tube.
**Figure S7**. Condensed survival data for the parental acclimation experiment. Each point represents the average difference in survival across replicate tubes for one parental couple.
**Figure S8**. Condensed survival data for the juvenile acclimation experiment. Each point represents the average difference in survival across replicate tubes for one parental couple.
**Figure S9**. Raw survival data for the microbiome experiment. Each point represents one replicate tube.
