# Supplementary material for: Phenotypic but no genetic adaptation in zooplankton 24 years after an abrupt +10°C climate change
Source: Evol Lett. 2022 Jul 6;6(4):284–94. doi: 10.1002/evl3.280 (PMC9346084; doi:10.1002/evl3.280)
Supplement: Supplementary file 1 — Table S1. Models used to generate effect sizes and variances for the meta‐analysis of phenotypic adaptation (solid points, Fig. 1A), which compared the overall temperature tolerance of VCH and SFB populations. Table S2. Significance of the tested effects for the individual experiments. Temp., temperature; treatm., treatment. Figure S1. Illustration of possible outcomes for the different experiments, with simple scenarios described next to the figures. Figure S2. Post‐hoc analysis of additive effects. In order to analyse the phenotypic change through time, we computed the slope of the log odd score through time (taking SFB84 , VCH97, and VCH08 as time 0, 1, and 2, respectively). Figure S3. SNP frequency data quality. SNP frequency was independently estimated twice for years 1984 (red), 1997 (orange), and 2008 (brown). Figure S4. Frequency of a strongly beneficial recessive allele (s = 0.3) through time in a population of N = 107 (panel A) or N = 106 (panel B) individuals. Figure S5. Survival data that was used in the meta‐analysis of phenotypic effect. Figure S6. Raw survival data for the additive effect experiment. Each point represents one replicate tube. Figure S7. Condensed survival data for the parental acclimation experiment. Each point represents the average difference in survival across replicate tubes for one parental couple. Figure S8. Condensed survival data for the juvenile acclimation experiment. Each point represents the average difference in survival across replicate tubes for one parental couple. Figure S9. Raw survival data for the microbiome experiment. Each point represents one replicate tube. [file EVL3-6-284-s001.docx]

|  |  | **T_SFB_** | **T_VCH_** |
| --- | --- | --- | --- |
| **Experiment** | **Fixed and random terms** | **Effect size ± SE** | **Effect size ± SE** |
| **Parental acclimation** | ~ *VCH population* + (1\|*Family*/*Clutch*^2^) | VCH_97_: -0.77 ± 0.27  VCH_08_: -0.30 ± 0.27 | VCH_97_: 0.35 ± 0.21  VCH_08_: 1.18 ± 0.19 |
| **Juvenile acclimation^1^** | ~ *VCH population* + (1\|*Family*/*Observation*) | VCH_97_: 0.38 ± 0.68  VCH_08_: -0.25 ± 0.81 | VCH_97_: 0.19 ± 0.46  VCH_08_: 0.85 ± 0.48 |
| **Microbiome** | ~ *VCH population* + (1\|*Observation*) | VCH_97_: -0.18 ± 0.28  VCH_08_: -0.06 ± 0.28 | VCH_97_: 0.52 ± 0.23  VCH_08_: 0.86 ± 0.22 |

**Table S1.** Models used to generate effect sizes and variances for the meta-analysis of phenotypic adaptation (solid points, Fig. 1A), which compared the overall temperature tolerance of VCH and SFB populations. Effect sizes are the log odds ratio of survival compared to SFB_84_. Where necessary, observation-level random effects were added to control for overdispersion. Notes: ^1^To estimate overall survival in this experiment, we fit models for survival in Phase 1 and Phase 2, then transformed, multiplied, and back-transformed the predicted survival rates. Standard errors were obtained by resampling. ^2^These models no longer have *Clutch* as a fixed term, so we control for possible variation in survival between the first and second clutches by including it here as a (non-dummy) random effect.

|  |  | **T_SFB_** | | **T_VCH_** | |
| --- | --- | --- | --- | --- | --- |
| **Experiment** | **Fixed-effect term** | **Test statistic** | ***P*** | **Test statistic** | ***P*** |
| **Additive genetic effects** | *Population* | *χ*^2^_(2)_ = 1.7 | 0.44 | *χ*^2^_(2)_ = 3.6 | 0.16 |
| **Juvenile acclimation** | *Population* | *χ*^2^_(2)_ = 28.8 | < 0.0001 | *χ*^2^_(2)_ = 44.4 | < 0.0001 |
|  | *Temp. in Phase 1* | *χ*^2^_(1)_ = 30.8 | < 0.0001 | *χ*^2^_(1)_ = 0.0 | 0.91 |
|  | *Population : Temp. in Phase 1* | *χ*^2^_(2)_ = 2.3 | 0.31 | *χ*^2^_(2)_ = 1.0 | 0.62 |
| **Parental acclimation** | *Clutch : Population* | *χ*^2^_(2)_ = 2.1 | 0.36 | *χ*^2^_(2)_ = 1.8 | 0.41 |
|  | *Clutch : Parental treatm.* | *χ*^2^_(2)_ = 3.3 | 0.19 | *χ*^2^_(2)_ = 3.8 | 0.15 |
|  | *Clutch : Population : Parental treatm.* | *χ*^2^_(4)_ = 8.3 | 0.08 | *χ*^2^_(4)_ = 5.6 | 0.23 |
| **Microbiome** | *Population* | *-* | - | *-* | - |
|  | *Microbiome* | *-* | - | *-* | - |
|  | *Population : Microbiome* | *χ*^2^_(4)_ = 15.7 | 0.003 | *χ*^2^_(4)_ = 18.4 | 0.001 |

**Table S2.** Significance of the tested effects for the individual experiments. Temp., temperature; treatm., treatment.

| Experiments | Illustration of possible outcomes in simple scenarios |
| --- | --- |
| Some possible outcomes for the parental acclimation experiment | 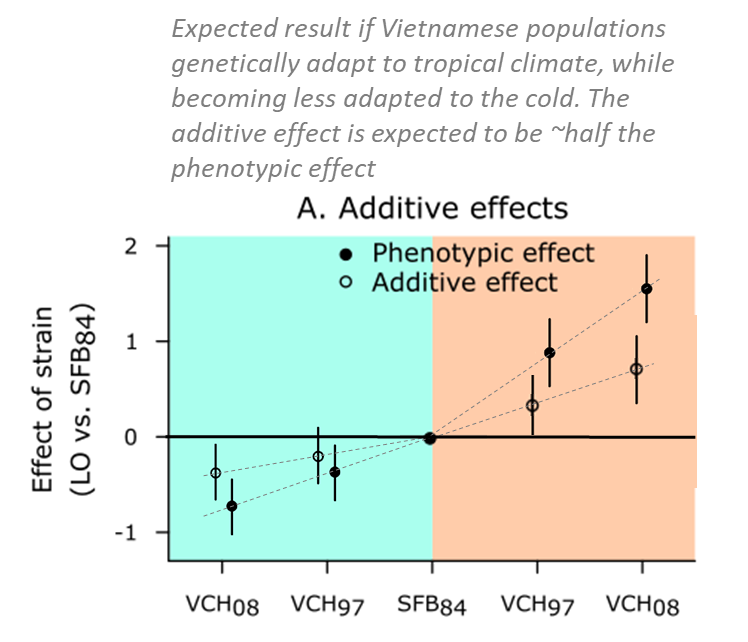 |
| Some possible outcomes for the parental acclimation experiment | 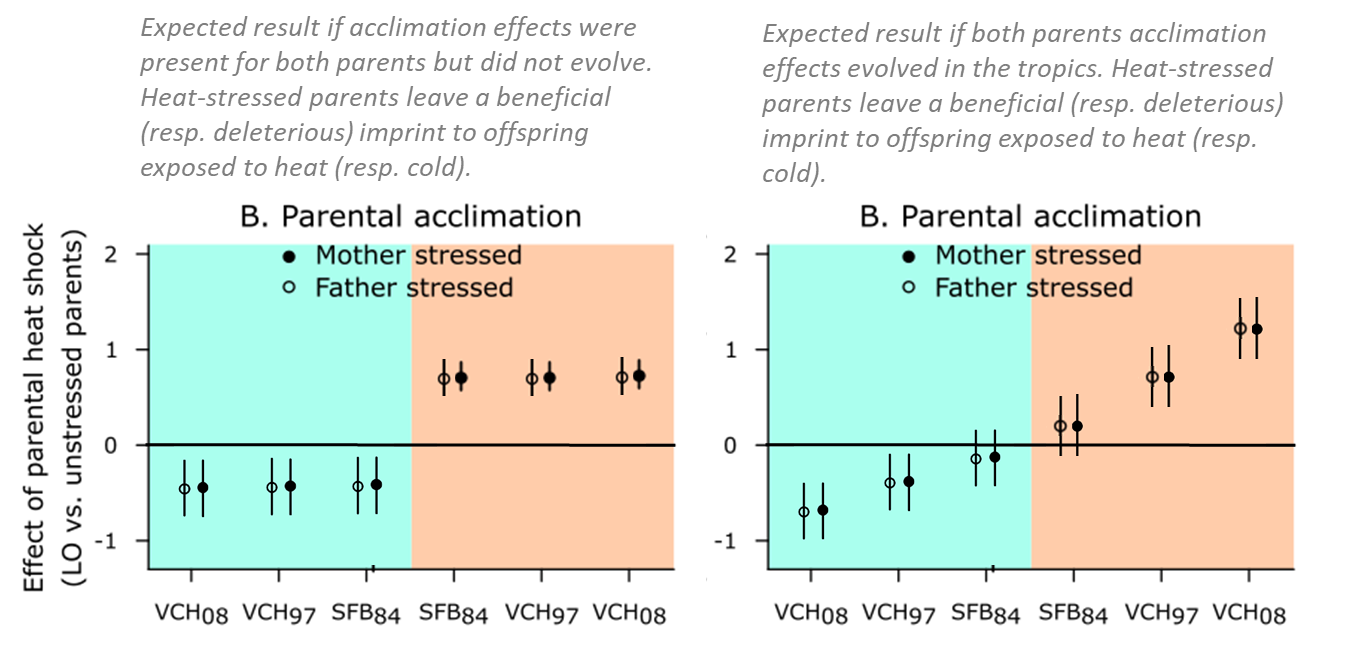 |
| Some possible outcomes for the juvenile acclimation experiment | 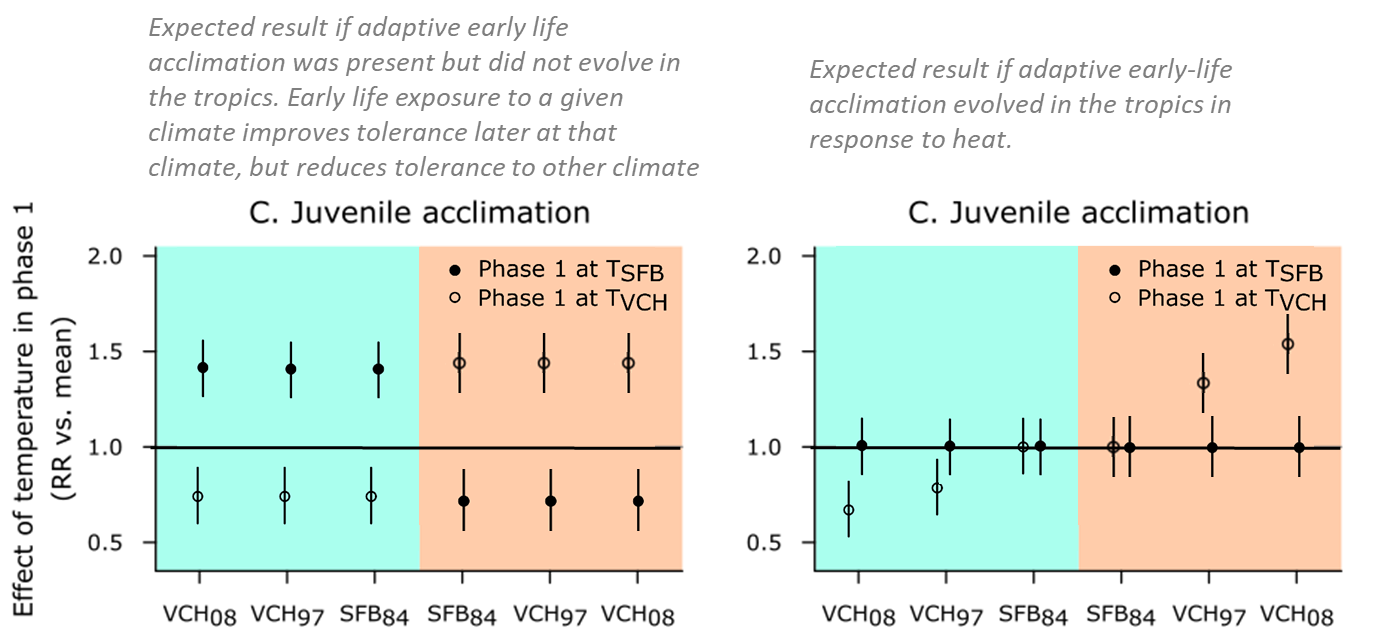 |
| Some possible outcomes for the microbiota experiment | 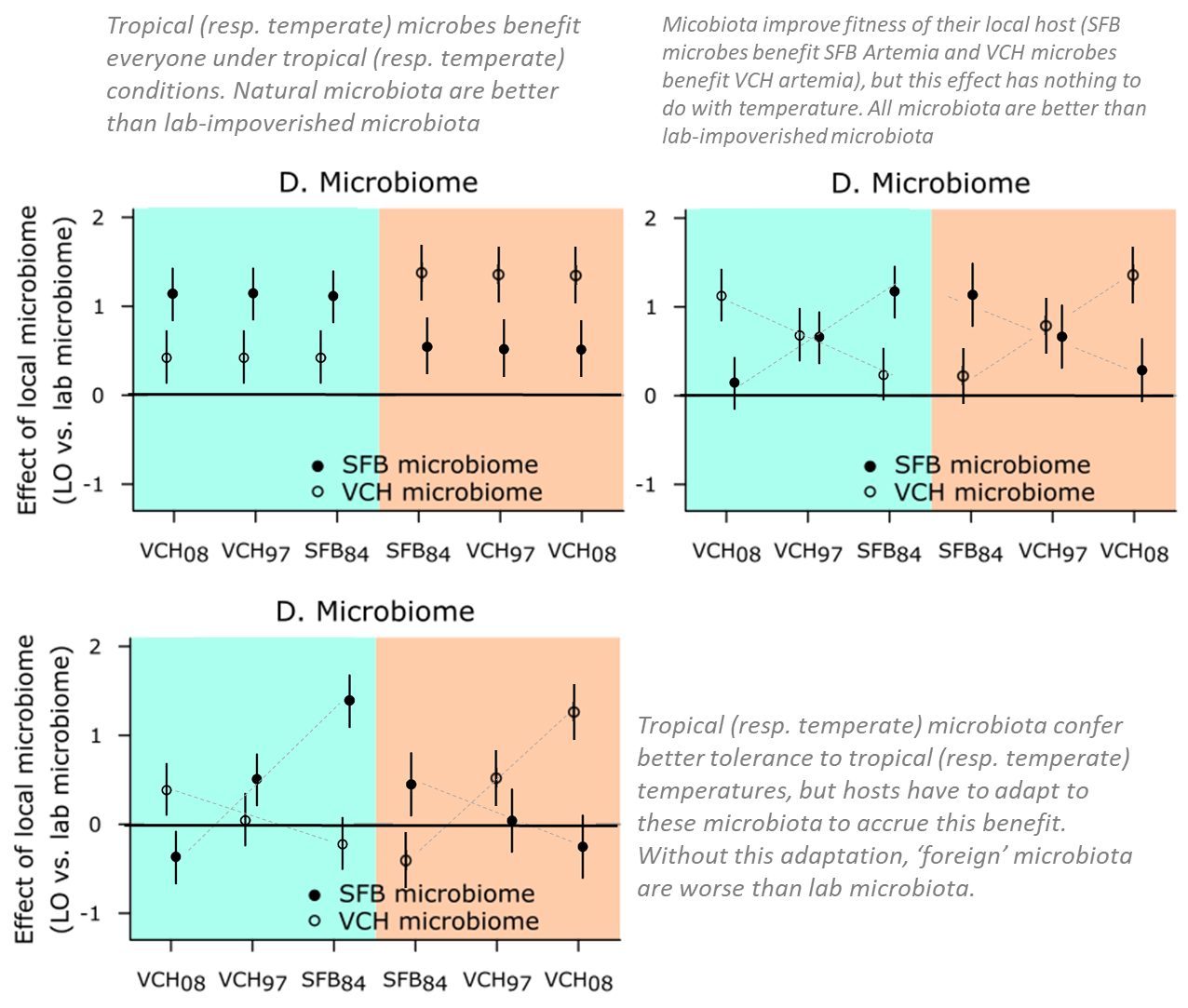 |

**Figure S1.** Illustration of possible outcomes for the different experiments, with simple scenarios described next to the figures**.**


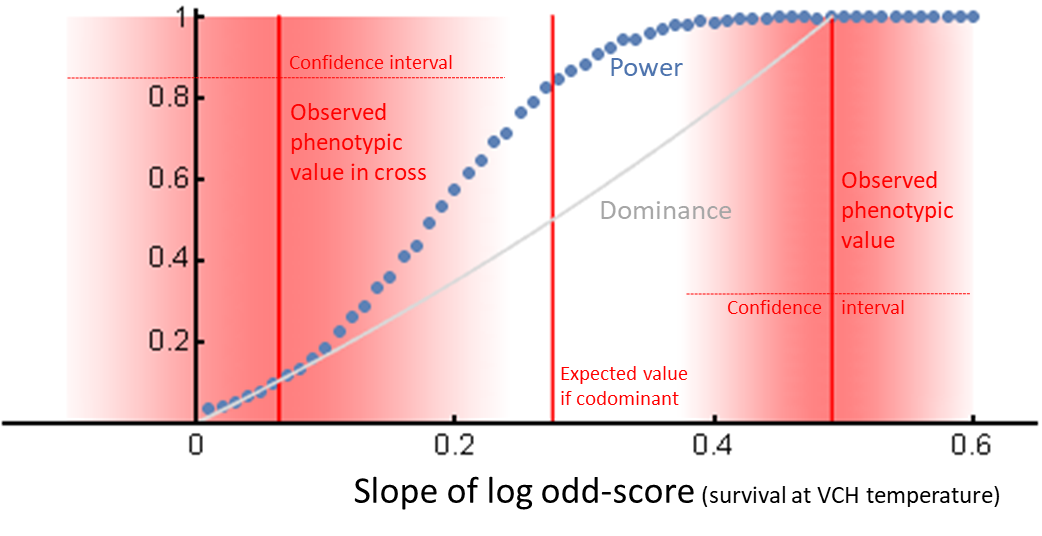


**Figure S2.** Post-hoc analysis of additive effects. In order to analyse the phenotypic change through time, we computed the slope of the log odd score through time (taking SFB_84_ , VCH_97_, and VCH_08_ as time 0, 1, and 2, respectively). We computed confidence intervals of this slope using resampling, assuming point estimates were normally distributed around their observed mean and with their measured standard deviation. This produced the confidence intervals around the observed phenotypic value and the observed phenotypic value in the cross (shaded in red). We also computed the chance of detecting a significant effect for different effect sizes, given our design and the observed standard deviations (the power, shown in blue). The grey line shows the phenotypic value expected in the cross (x-axis) given a certain dominance level (value on the y-axis). This analysis shows that additive effects (resulting in codominance) can be excluded, and that if the change in thermal tolerance had a (nuclear) genetic basis (rather than an epigenetic or mitochondrial one), it would be strongly, but perhaps not fully, recessive (the point estimate is *h* = 0.10 as shown on the figure). For the expected response to selection with this dominance level, see fig. S4.


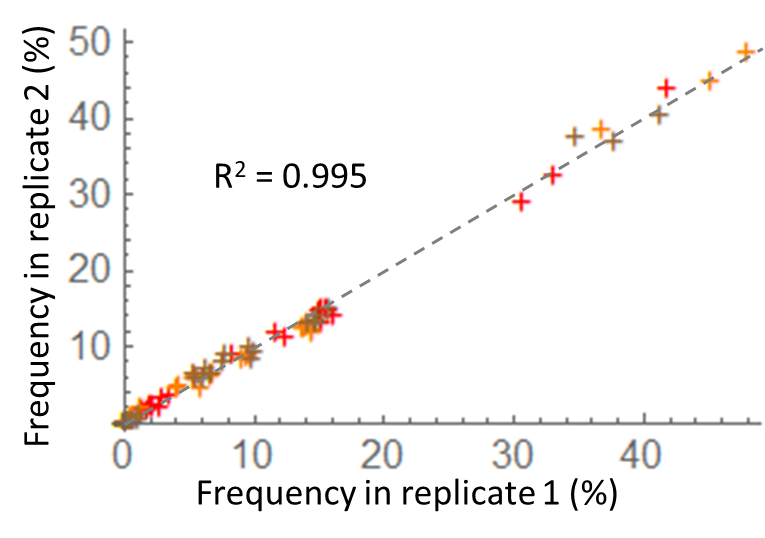


**Figure S3.** SNP frequency data quality. SNP frequency was independently estimated twice for years 1984 (red), 1997 (orange), and 2008 (brown). The figure reports the correlation between these replicated values for all SNPs used in Fig. 2 (all private and shared SNPs among sequenced individuals). R^2^ = 0.995 over all replicated measures. Dashed line is the 1:1 line.


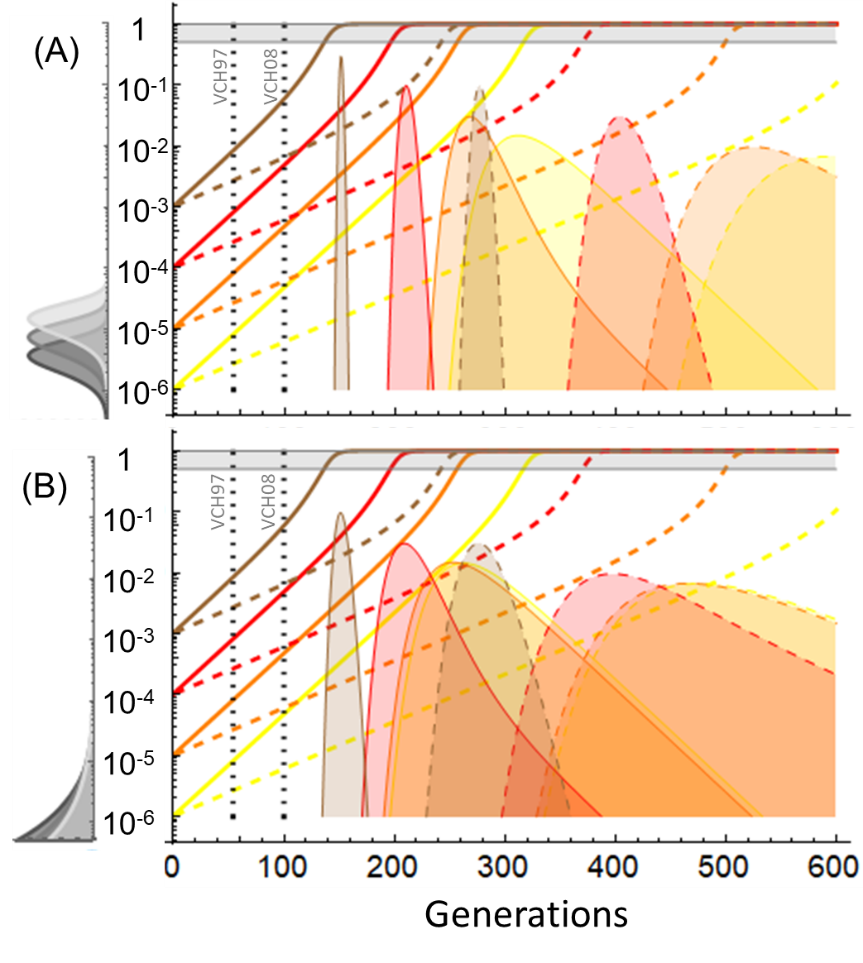


**Figure S4**. Frequency of a strongly beneficial recessive allele (*s* = 0.3) through time in a population of *N* = 10^7^ (panel A) or *N* = 10^6^ (panel B) individuals. Plain and dashed lines: exact deterministic frequency change for this beneficial allele given a dominance coefficient of *h* = 0.13 and *h* = 0.06, respectively. For these curves, the *x*-axis represents time in generations, and the *y*-axis is the frequency in log-scale. Different colors illustrate different initial frequencies, from 10^-6^ (yellow) to 10^-3^ (brown). For comparison, a newly arising mutant would start at 10^-7^ or 10^-6^ in panel A and B, respectively. The horizontal gray bar represents the [0.5,1] frequency range for the beneficial allele, within which it starts having a detectable effect on the mean fitness of the population (~10% increase in mean fitness). The vertical dotted lines correspond to our two dates of measurement (1997, at generation ~54, and 2008, at generation ~100). Colored surface areas show the corresponding probability density of half-sweep times (i.e. the number of generations to reach a frequency of ½) from a stochastic model with *N_e_* = *N* (from Eq. 8 in (Martin and Lambert 2015), same line and color code for dominance and initial frequency as for the deterministic curves). Here, the *x*-axis shows this half-sweep time and the *y*-axis the corresponding probability density. Distributions in grey to the left of the *y*-axis show the frequency distribution of the heat tolerance allele in its population of origin (SFB), at mutation (*u* = 10^-7^) selection balance, assuming that it reduces fitness at cold temperatures by a modest amount (*hs* = 0.02 dark grey, 0.01 grey or 0.005 light grey) compared to its strong advantage at high temperature. This Wright’s distribution is computed from Eq. 9.3.4 in (Crow and Kimura 1970) with *N_e_* = *N*. *y*-axis: frequency, *x*-axis: probability density (rescaled for readability on panel A by the inverse of the density at mean frequency). Overall, the figure shows that even in the most favorable conditions, adaptation caused by a recessive beneficial allele cannot be detected within the short 54 or 100 generations of our study. These are the most favorable conditions, as we consider (1) incompletely recessive beneficial alleles, with *h* = 0.13 corresponding to the point estimate based on observed survival in our experiment 1 for VCH_08_ which is certainly a maximum given that the point estimate in VCH_97_ would be *h* = 0 at most; and the compound estimate is *h* = 0.10, see Fig S2); (2) a very strong beneficial effect *s* = 0.3, comparable to the highest published field estimates of fitness effects in situations of intense selection pressures (e.g. insecticide resistance Lenormand et al. 1999); (3) plausible population sizes of *Artemia* populations, as census sizes in the field far exceed 10^6^ in Vinh Chau saltern; (4) a modest deleterious fitness effect of these beneficial alleles at cold temperature and standard mutation rate (10^-7^; a ten-fold larger mutation rate would not alter the conclusion, so that these mutations are unlikely to segregate at a higher frequency than 10^-3^ in the population of introduction). In addition, with such intense selection, beneficial alleles spread extremely fast when they reach a frequency above 0.2 (they then reach a frequency of 0.8 in ~20 generations), so that in this scenario, with a strongly beneficial allele we would be likely to observe a fitness change only between 1984 and 1997 or 1997 and 2008, but not both.


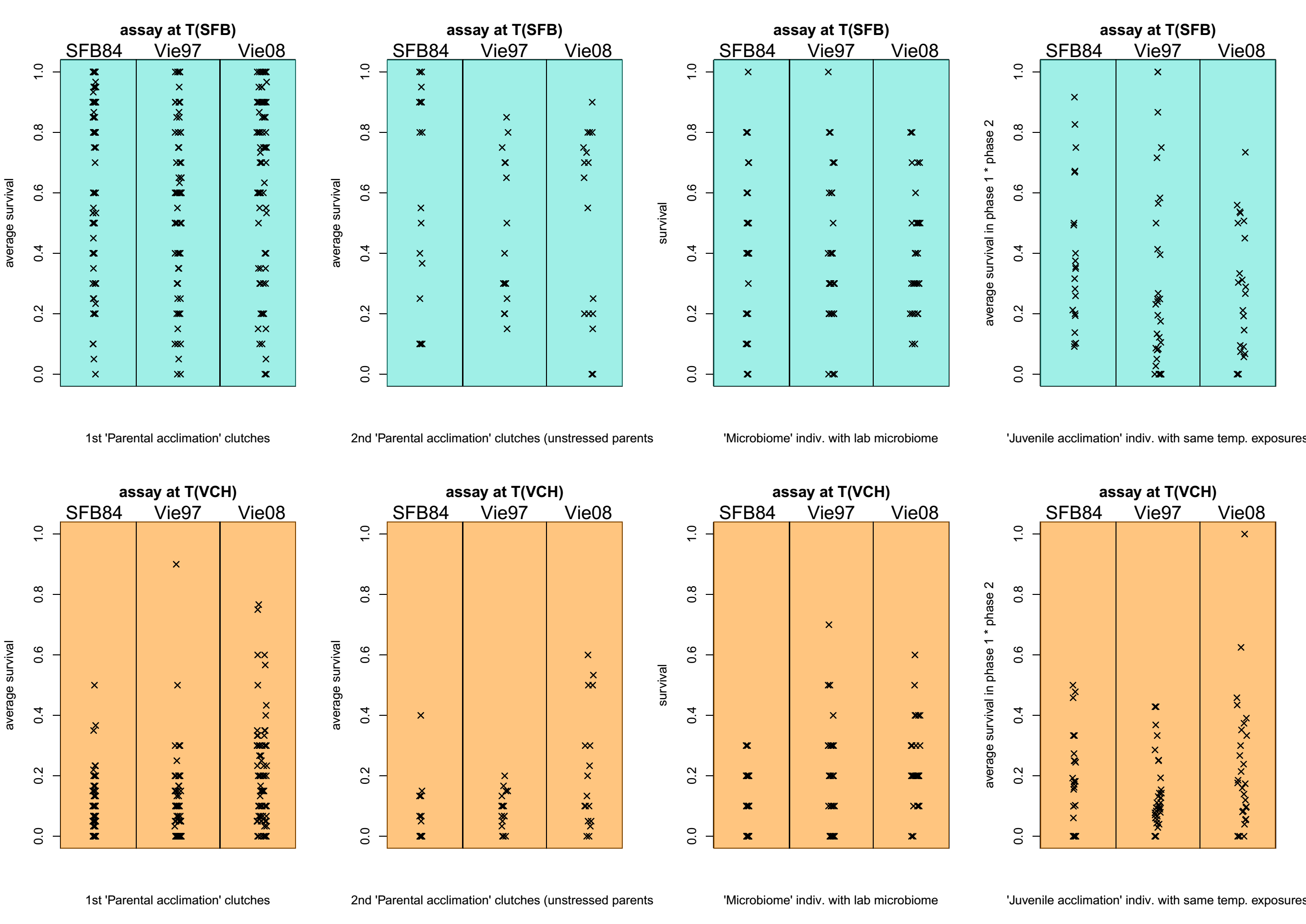


**Fig. S5** Survival data that was used in the meta-analysis of phenotypic effect. Each point represents one replicate tube (‘Microbiome’ plots) or the average survival across replicate tubes for one parental couple (all others).


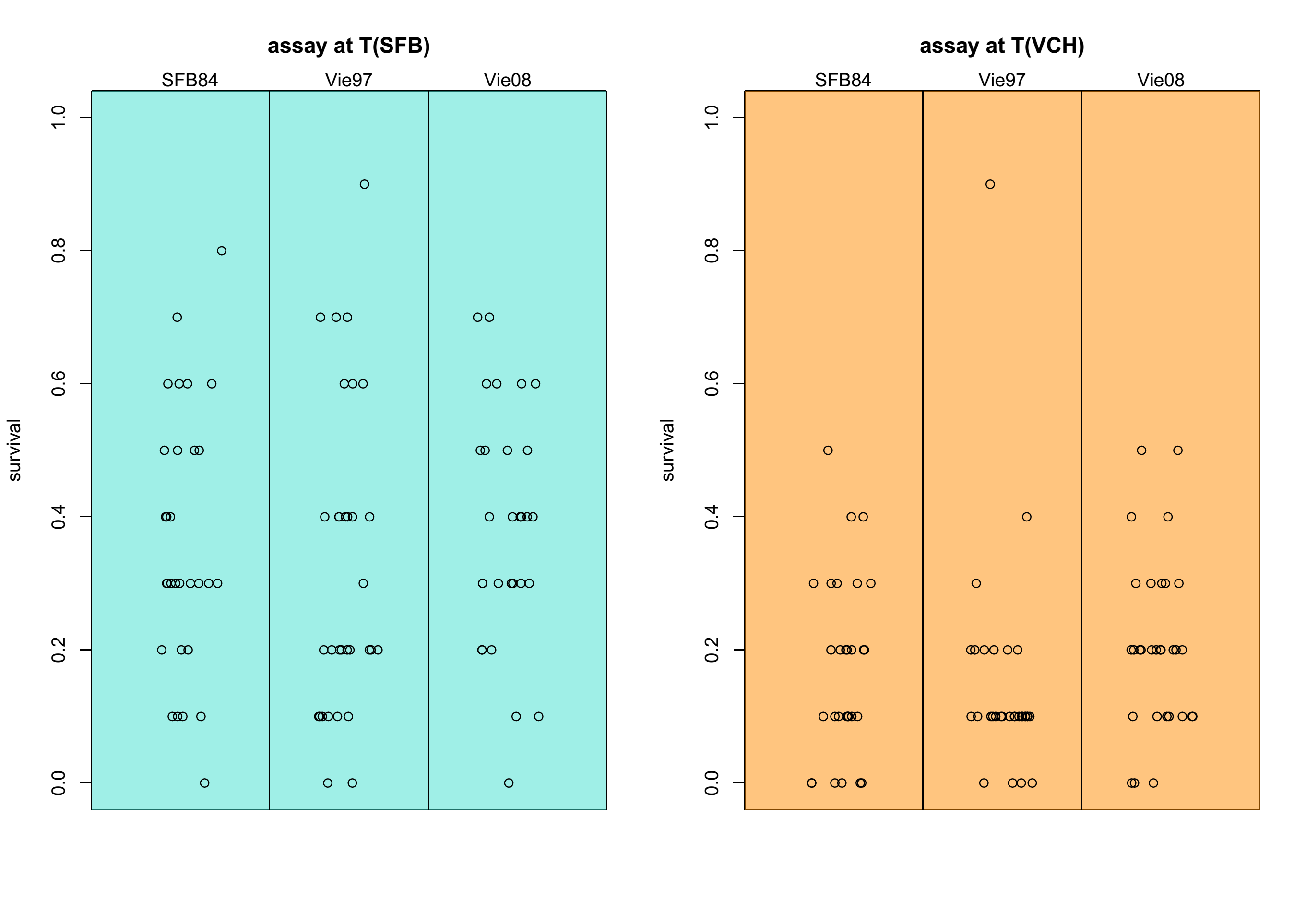


**Fig. S6** Raw survival data for the additive effect experiment. Each point represents one replicate tube.


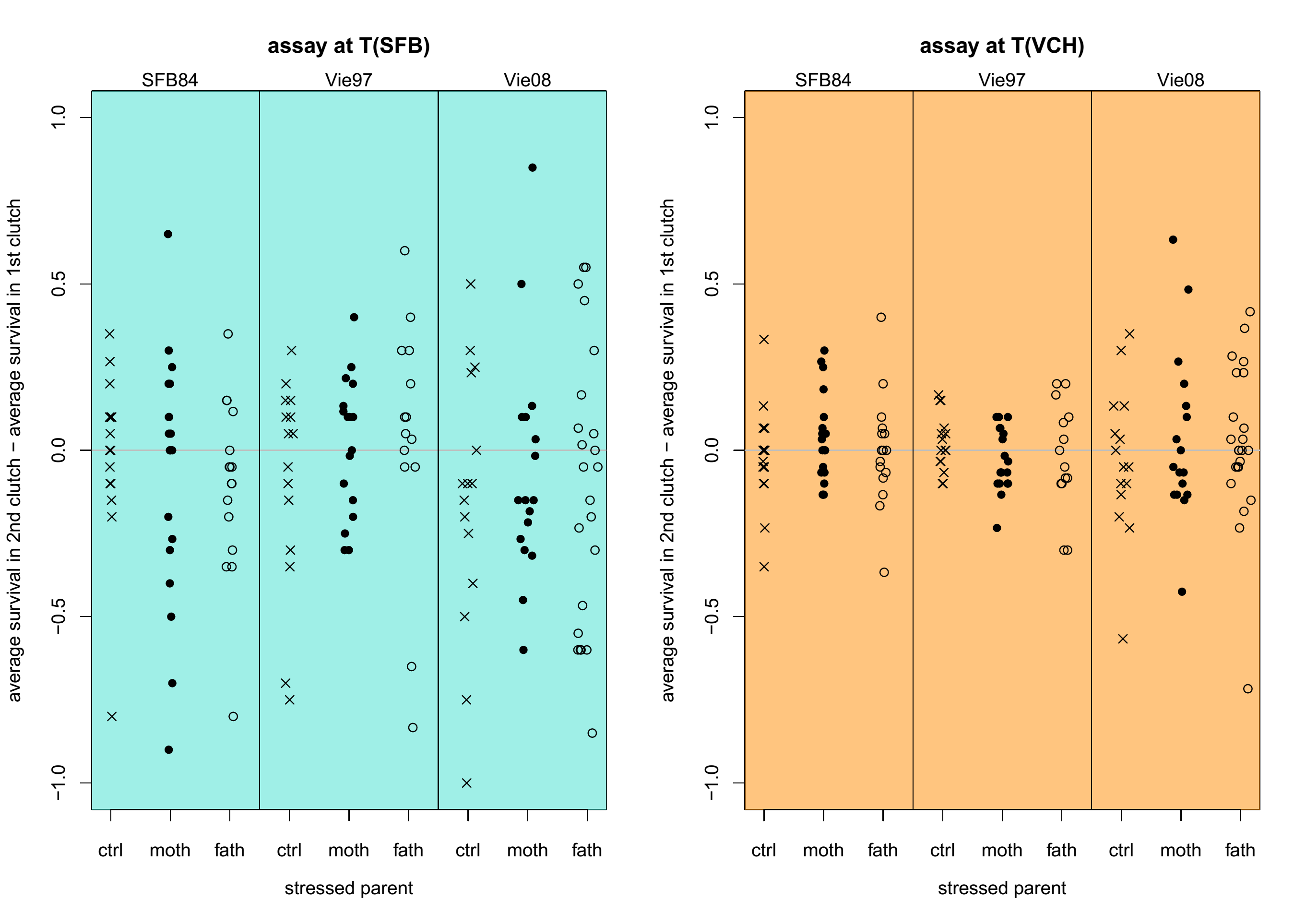


**Fig. S7** Condensed survival data for the parental acclimation experiment. Each point represents the average difference in survival across replicate tubes for one parental couple.


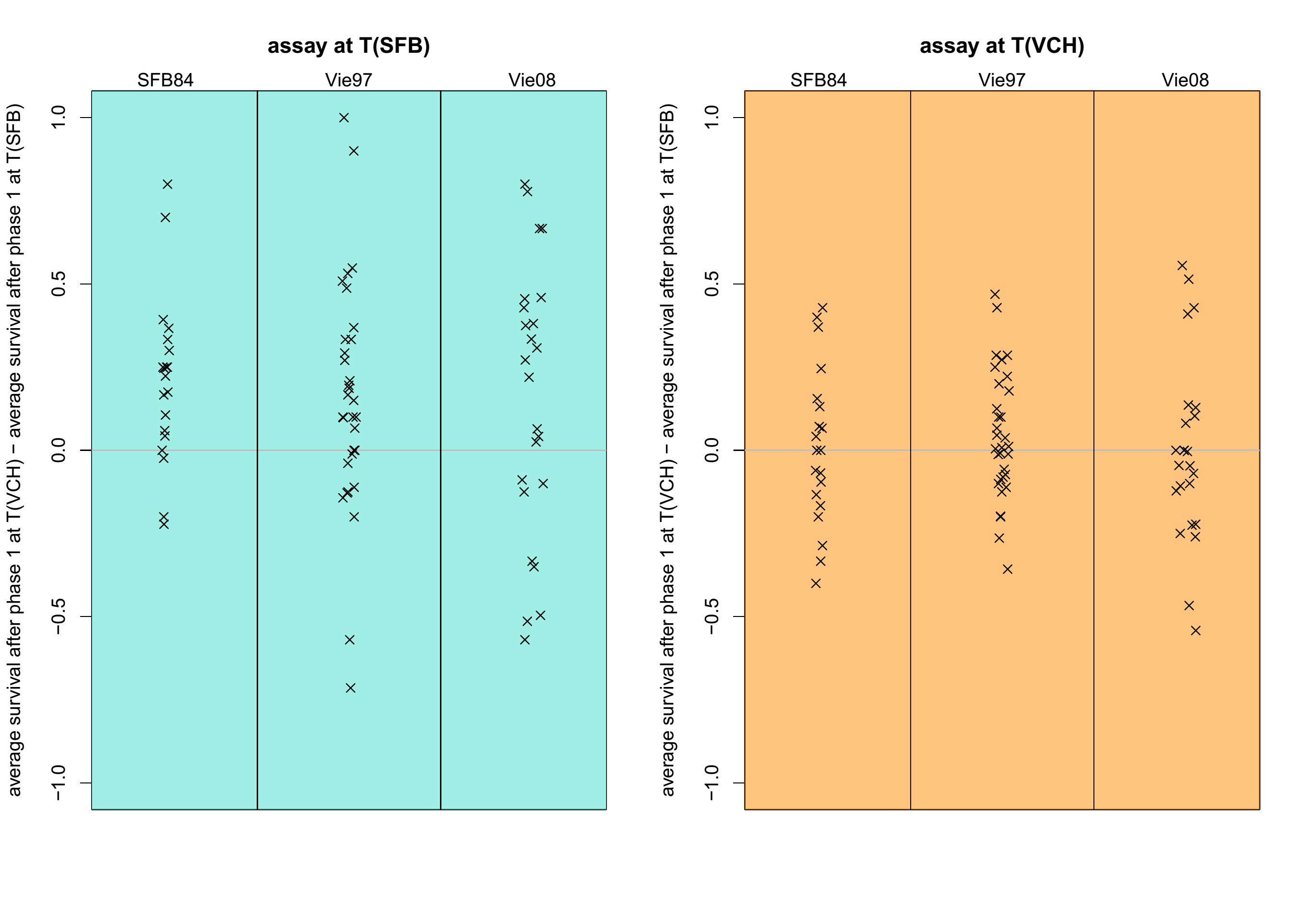


**Fig. S8** Condensed survival data for the juvenile acclimation experiment. Each point represents the average difference in survival across replicate tubes for one parental couple.


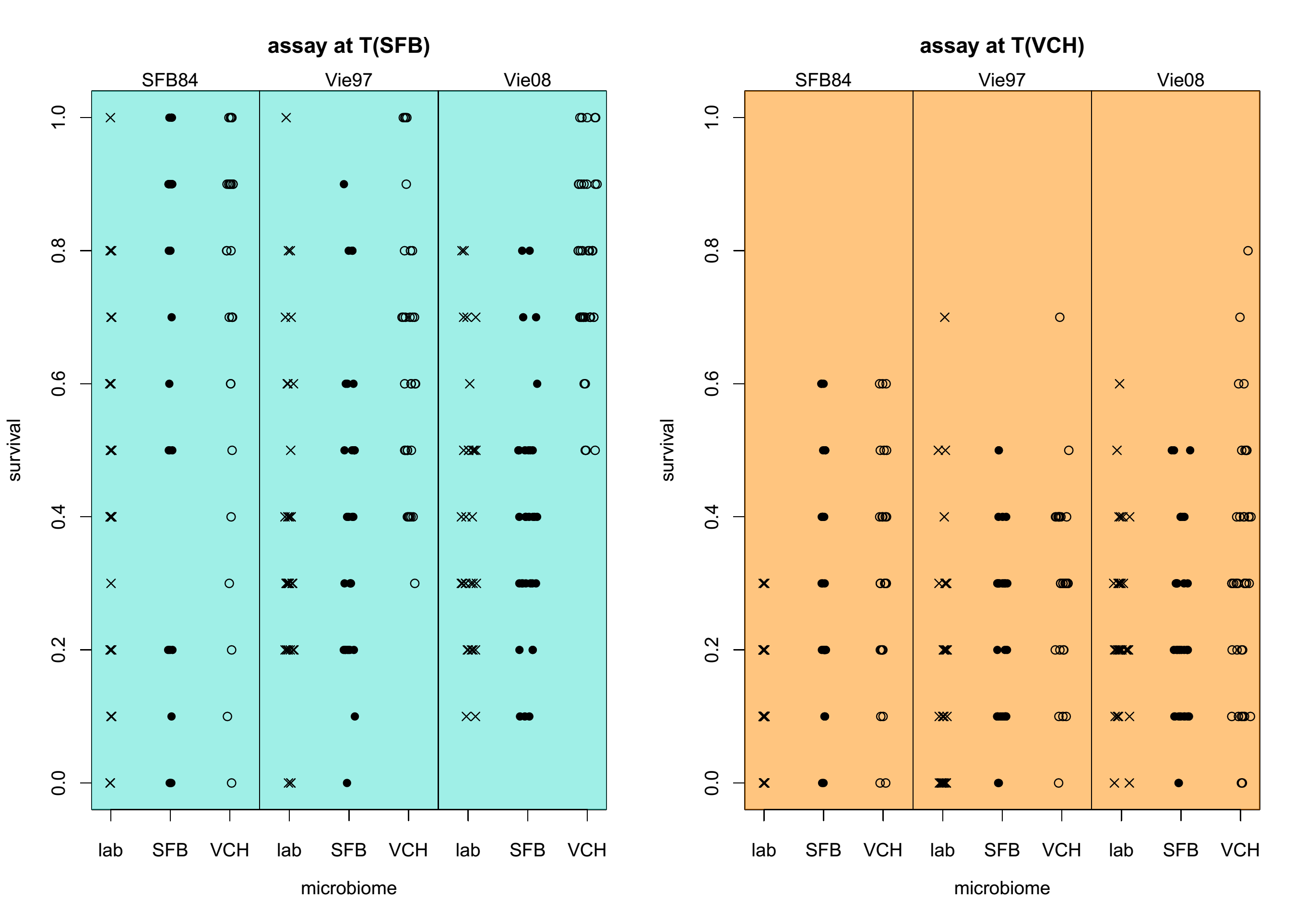


**Fig. S9** Raw survival data for the microbiome experiment. Each point represents one replicate tube.
